# Computational Molecular Nanoscience Study of the Properties of Copper Complexes for Dye-Sensitized Solar Cells

**DOI:** 10.3390/ijms131216005

**Published:** 2012-11-28

**Authors:** Jesús Baldenebro-López, José Castorena-González, Norma Flores-Holguín, Jorge Almaral-Sánchez, Daniel Glossman-Mitnik

**Affiliations:** 1 Centro de Investigación en Materiales Avanzados, S.C., Miguel de Cervantes 120, Complejo Industrial Chihuahua, Chihuahua 31190, Mexico; E-Mails: jesus.baldenebro@cimav.edu.mx (J.B.-L.); norma.flores@cimav.edu.mx (N.F.-H.); 2 Universidad Autónoma de Sinaloa, Prol. Ángel Flores y Fuente de Poseidón, S.N., Los Mochis, Sinaloa 81223, Mexico; E-Mails: kstor28@yahoo.com.mx (J.C.-G.); jalmaral@gmail.com (J.A.-S.)

**Keywords:** molecular structure, absorption spectra, polarizability, chemical reactivity, dipole moment, copper complex, dye-sensitized

## Abstract

In this work, we studied a copper complex-based dye, which is proposed for potential photovoltaic applications and is named Cu (I) biquinoline dye. Results of electron affinities and ionization potentials have been used for the correlation between different levels of calculation used in this study, which are based on The Density Functional Theory (DFT) and time-dependent (TD) DFT. Further, the maximum absorption wavelengths of our theoretical calculations were compared with the experimental data. It was found that the M06/LANL2DZ + DZVP level of calculation provides the best approximation. This level of calculation was used to find the optimized molecular structure and to predict the main molecular vibrations, the molecular orbitals energies, dipole moment, isotropic polarizability and the chemical reactivity parameters that arise from Conceptual DFT.

## 1. Introduction

The current warming of the global climate is the result of an increase in greenhouse gas (GHG) emissions, particularly CO_2_. Global average atmospheric CO_2_ has increased from 280 ppm in the 1750s to 389 ppm in 2010 [1–3]. An increasing demand for energy in the emerging economies and energy crisis worldwide has stimulated a growing number of researches on renewable energy, in that the utilization of renewable energy can help reduce fossil fuel consumption and alleviate environmental problems. Renewables-based power systems provide an opportunity to generate cleaner electricity with a lower cost of energy [4]. It is thought that the transition from fossil fuels to a diversified energy matrix can be accelerated by governments by means of adequate policies and instruments that support the creation of incentives for mitigation of greenhouse gases (GHG) emissions and investments in renewable energy technology research and development. Solar energy is one of the most promising sources of energy in the future and one of the renewable energy resources that has long played a dominant role in the field of energy research with its wide application and great potential [5,6]. Recently, dye sensitized solar cells (DSSC) [7–9], considered as a credible alternative to conventional inorganic silicon-based solar cells, have attracted attention due to their efficiency, simple manufacturing and low cost [10–13]. In these DSSC, an organic sensitizer must be chemically absorbed on the porous surface of the nanocrystalline oxide. After absorbing a photon, the excited electron in the dye-sensitized molecule is transferred into the conduction band of nanocrystalline oxide, followed by a process in which the electron diffuses through the electrode. The sensitizer in this oxidized state is reduced to its normal state gaining electrons through a liquid electrolyte [14–16]. Nowadays, many research groups from all over the world actively participate to improve the efficiency of every single process involved in the DSSC [17–19]. The charge transfer efficiency from the dye molecule to the nanocrystalline oxide is extremely important in the solar cell design. Since Regal and Grätzel published their pioneer study [7], the understanding of the mechanism has required fundamental research about the diverse physical phenomena at nanometric scale [20]. Theoretical studies on physical and chemical properties of dye-sensitizers are very important to understand the relationship between the structure, properties and performance in order to design and synthesize new molecules for this purpose [21–24].

**Figure 1:**
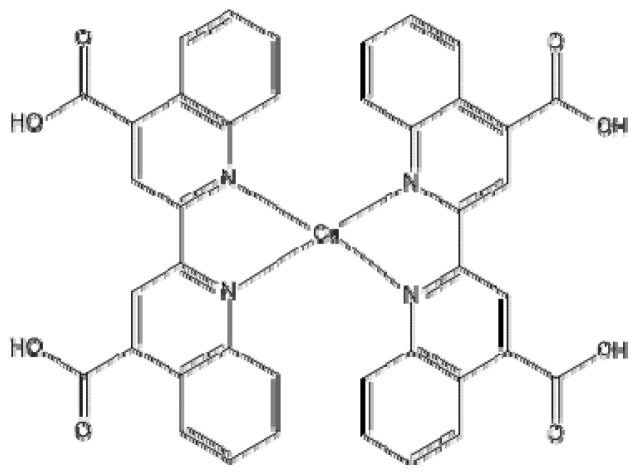
Molecular structure of copper complex.

Ruthenium(II) complexes as dyes have been extensively used as sensitizers in DSSCs owing to their strong absorption in the visible range and relatively long-lived excited states [25]. The strong absorptivity of these complexes is due to a metal-to-ligand charge transfer (MLCT) transition [26]. These complexes have reached over 12% power conversion efficiency [27]; but the rarity and high cost of the Ru may limit their practical usage. Since initial reports of a [Ru(bpy)_3_]^2+^-based (bpy = 2,2′-bipyridine) photosensitizer, the dyes reported in the literature have predominantly been ruthenium(II) complexes. We have recently become interested in the study and optimization of copper(I)-based DSSC, literature reports of which are scarce. Sauvage and co-workers [28] discovered that copper(I) complexes have similar photo-physical properties with Ru complexes, indicating that the iterative chemical optimization of common metal complexes sensitizers can be comparable to that of Ru complexes [29]. Copper(I) complexes display a wide variety of excited states and especially photophysical and photochemical processes. Particularly, copper(I)–poly-pyridine complexes exhibit low-lying MLCT transitions that can participate, among others, in electron transfer processes [30]. In this research, we propose the study of a molecular system of this type, such as [Cu(LL)_2_]^+^ (LL = 2,2′-biquinoline-4,4′-dicarboxylic acid) which is shown in Figure 1 (Cu(I) biquinoline), in order to define from the theoretical point of view a suitable calculation methodology for obtaining structural parameters, as well as electrical and optical properties using the density functional theory (DFT) [31–33] and time-dependent (TD) DFT [34–36]. These methods are implemented in the Gaussian 09W program package [37]. In this paper, we have found very interesting properties with the proposed ligand to increase the level of conjugation, which has not been reported as an article in the DSSC field.

## 2. Results and Discussion

Once the molecular structure was proposed, the geometry optimization was calculated, followed by the frequency analysis to confirm that the species had the minimum energy conformation. These calculations were performed in the presence of methanol as solvent.

The electron affinity (A) and the ionization potential (I) were obtained by energy calculations (neutral and ionic state), taking into account the ground state geometry optimization. The aim of this was to establish the correlation of results between the different levels of calculation, since there are no experimental results reported for this system; this is a contribution of our research. Table 1 shows the values obtained and where it seems as if some of these have variations between them.

**Figure 2:**
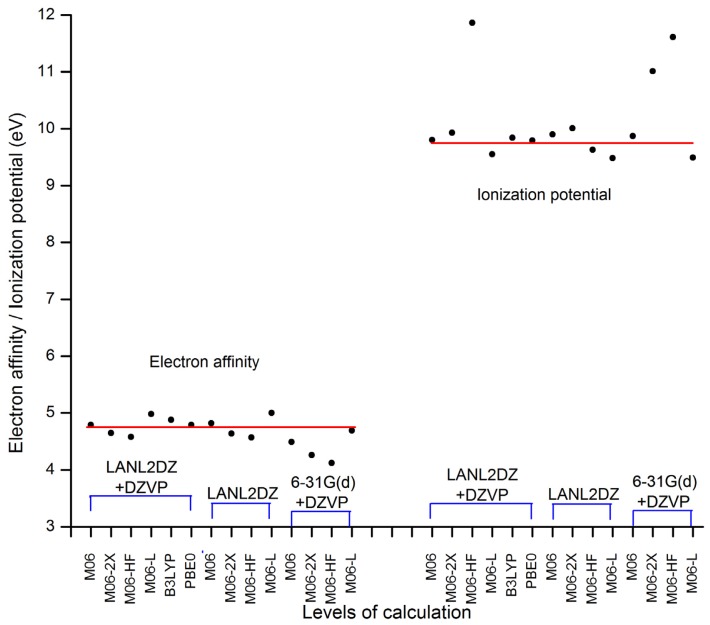
Electron affinity and ionization potential for the levels of theory used in this study and the dispersion between the values. The red line represents the mean value, in the case of electron affinity it is located at 4.75 eV and in the case of ionization potential it is equal to 9.75 eV.

Figure 2 helps to visualize more clearly the dispersion of these values. M06-HF/LANL2DZ-DZVP, M06-2X/6-31G(d) + DZVP and M06-HF/6-31G(d) + DZVP levels of theory show the greatest dispersion; therefore, the values are ignored for the calculation of some measures of central tendency, such as the mean and median. The red line represents the mean value, in the case of electron affinity, it is located at 4.75 eV and for the ionization potential it is equal to 9.75 eV; meanwhile, the medium has the values of 4.79 eV and 9.80 eV, respectively. This led to results of the population standard deviation of 0.1555 (in A) and 0.1768 (in I) with a coefficient of variation of 3.27% and 1.81%, which are values of high quality. On this basis, we can establish that the model chemistry M06/LANL2DZ + DZVP and PBE0/LANL2DZ + DZVP represent excellent approximations in this context.

Another fundamental property as a potential sensitizer for DSSC is the maximum absorption wavelength (λ_max_); according to the levels of theory selected, the UV-Vis spectrum and the λmax were calculated. The results of λ_max_ are shown in Table 2.

The results in Table 2 show that λmax varies from a high value to a low value when the functional increases the percentage of Hartree-Fock exchange. The experimental result indicates that λmax is 553 nm [38]. Comparing this result with our calculation, we can conclude that the calculation methodology with more precision is M06/LANL2DZ + DZVP. Similarly, by comparing the results of A, I and λmax, this methodology will be used to study other properties of the dye under investigation. Another aspect to consider is that using a LANL2DZ basis set better describes the behavior of the excited states of the ligand molecule.

**Figure 3:**
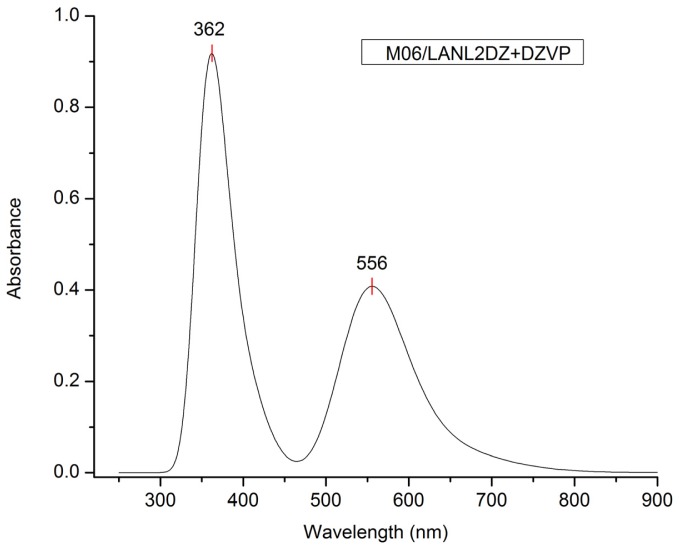
UV-Vis spectra of Cu(I) biquinoline dye at the M06/LANL2DZ-DZVP level of calculation.

Figure 3 shows the UV-Vis spectra at the M06/LANL2DZ-DZVP level of calculation and its corresponding λmax (556 nm). The calculated value of λmax indicates that this molecular system should be considered for use as a functional material (as dye in this case) in a DSSC, the value of this parameter for the Cu(I) biquinoline dye meets the requirements established in the literature [39].

Table 3 shows the result of time-dependent density functional theory (TD-DFT) calculation; including the corresponding wavelengths (in nm), the energies (in eV), the oscillator strength (f) and the orbitals involved in the transitions. This information shows that the peak of the wavelength of maximum absorption (556 nm) is due to charge transfer between the metal atom and the ligand molecule (MLCT). The spectrum of Cu(I) biquinoline also showed intense absorption in the UV region assigned to ligand-based π*—π transition (362 nm).

There are theoretical studies on copper complexes reported by X. Lu *et al.*[40], they used the B3LYP functional with 6-31G(d) and DZVP basis sets and obtained a value of λ_max_ equal to 543 nm; compared with the experimental result of 492 nm by E. Constable *et al.*[41], we can note the good approximation of this level of calculation. However, the proposed methodology with the M06 functional is more suitable due to lower error.

In their recent study on copper complexes, E. Constable *et al.*, 2010 [42] propose to incorporate substituents at the 6-6′ positions with imine-based ligands to protect the metal center and modulate the redox properties. The reached value of λ_max_ is 511 nm, which is less than for the system proposed in this study.

**Figure 4:**
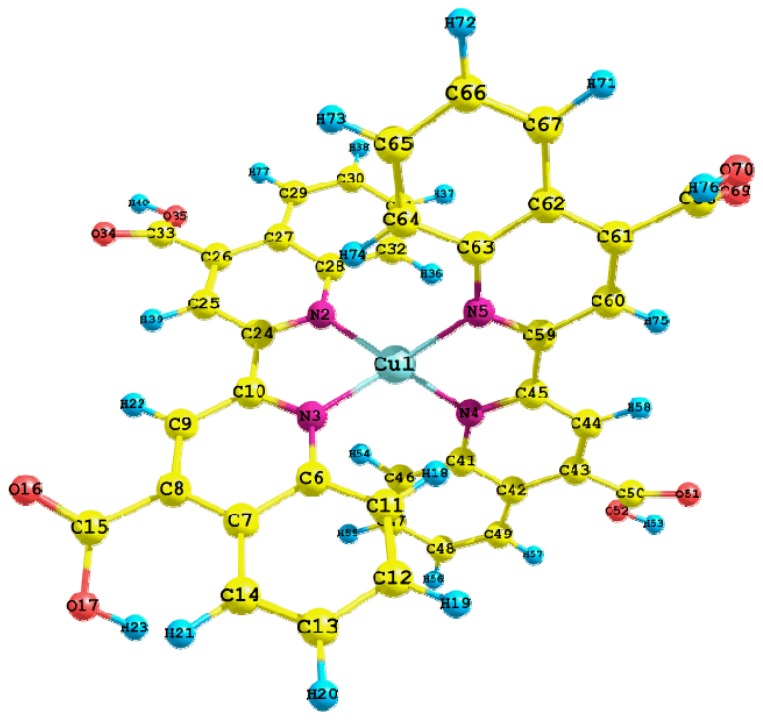
Optimized molecular structure of Cu(I) biquinoline dye with M06/LANL2DZ + DZVP level of calculation.

The optimized structure of Cu(I) biquinoline dye is shown in Figure 4, including the numbers of atoms and symbols. This geometry was obtained in the presence of methanol as solvent and the integral equation formalism polarizable continuum *model* (IEF-PCM) model.

A selection of geometric parameters was made to clearly visualize how the geometric structure has a very small structural variation of the atoms bonded with respect to the characteristic lengths and angles reported in the literature [28]. Table 4 shows the selected values for bond length (Å) and bond angles (in degrees).

Another very interesting research by X. Lu *et al.* 2011 [43] presents the molecular structures of eight copper complexes which display the results for bond lengths of 1.987 to 2.002 Å between the copper and nitrogen atoms, the bond angles vary from 82.8° to 83.2°. These values are very close to the typical ranges of 2.003 Å to 2.039 Å and 80.93° to 81.21°, which were reported by T. Bessho *et al.* in 2008 [41]. Considering the Table 4, the calculated values vary from 2.022 Å to 2.025 Å, while the bond angles are estimated at 81.6°. This shows the excellent approximation on the level of theory.

**Figure 5:**
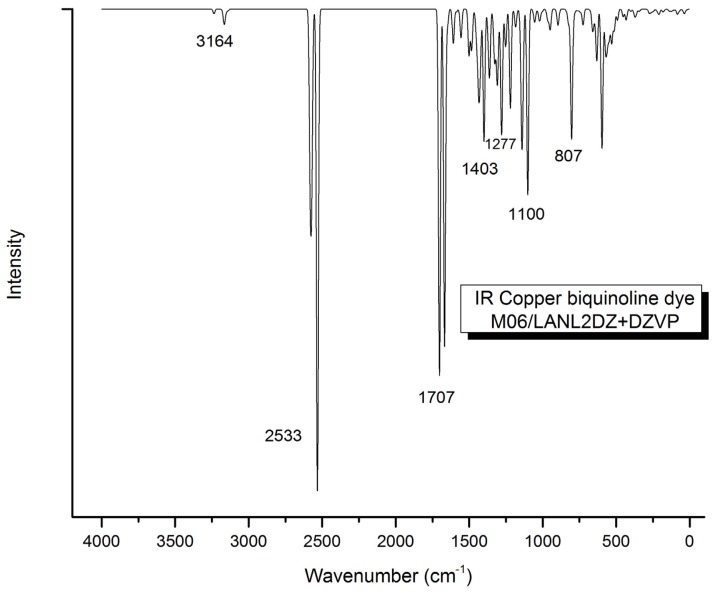
Infrared spectra of Cu(I) biquinoline dye.

In infrared (IR) spectral calculation (Figure 5), the vibrational bands indicate the presence of C–H, O–H, C=O and C–C bonds. The stretching vibration of the aromatic ring C–H bond is observed at 3164 cm^−1^. The O–H bond stretching vibration appears at 2533 cm^−1^, while another peak due to the vibration of C=O is present at 1707 cm^−1^. The vibration of the C–C bonds occurs at 1403 cm^−1^ and the bending of C–H in the rings is shown at 1277 cm^−1^. At 1100 cm^−1^, the corresponding vibration appears for the bending of the O–H bond. The out-of-plane bending vibration associated with the aromatic rings is observed at 807 cm^−1^. All signals are described in the typical ranges. This calculation, besides checking the minimum energy structure, also helps to elucidate the infrared spectra because this has not been reported in the literature up to now.

**Figure 6:**
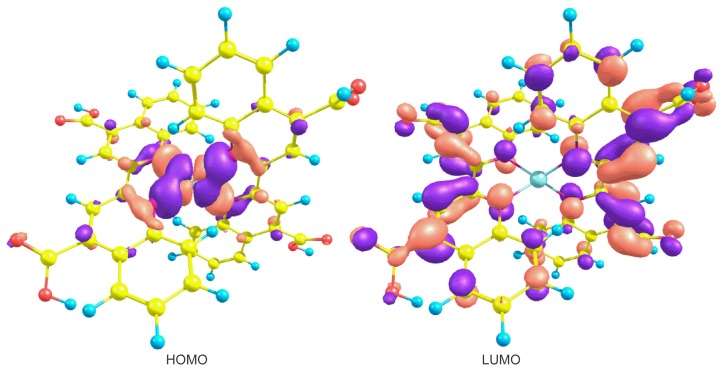
Highest occupied molecular orbitals (HOMO) and lowest unoccupied molecular orbitals (LUMO) orbitals of Cu biquinoline dye with M06/LANL2DZ + DZVP level of calculation.

The charge transfer efficiency from the dye molecule to the nanocrystalline oxide is extremely important in the solar cell design. Since the crucial electronic excitations occur from the highest occupied molecular orbitals (HOMO) to the lowest unoccupied molecular orbitals (LUMO), it is therefore important to form efficient charge-separated states with the HOMO localized on the donor subunit and the LUMO on the acceptor subunit. In this molecular system, Figure 6 shows that the HOMO orbital density is located around the copper atom; meanwhile, the density of the LUMO orbital is in the ligand and tends to move toward the carboxyl groups. This is beneficial in accordance with the above-mentioned.

**Figure 7:**
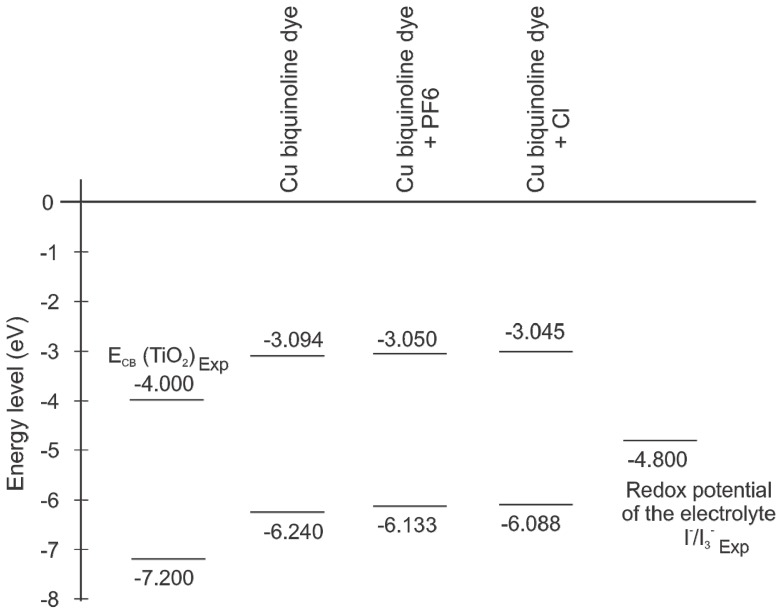
Molecular orbital energy level diagram.

The HOMO-LUMO molecular orbitals’ energetic position is another factor to consider, as the dye LUMO level must be greater than the conduction band level of nanocrystalline oxides that are commonly used in such devices [43]. Furthermore, the dye HOMO level is less than the redox potential of the electrolyte. The value found for LUMO level is −3.094 eV and for the HOMO level −6.240 eV (Cu complex cation). We also calculated the influence of anion species considering hexafluorophosphate (PF6^−^) and chlorine (Cl^−^). The diagram in Figure 7 illustrates how little activity these species contribute to the energy levels and that these levels meet the requirements. The band gap of Cu(I) biquinoline dye has a value of 3.146 eV, which is a value suitable for its consideration as a potential application in photovoltaic devices.

The values of the total dipole moment and the isotropic polarizability at the fundamental state obtained at the M06/LANL2DZ + DZVP level of calculation are 5.63 debye and 838.56 bohr^3^, respectively. Chemical reactivity parameters were obtained by energy calculations (neutral and ionic state), taking into account the ground state geometry optimization. These parameters are the electronegativity (χ = 7.30 eV), chemical hardness (η = 2.50 eV) and electrophilic index (ω = 10.64 eV). These results are of great importance, since they can be used during synthesis to determine the solubility and chemical reactivity of the molecule, and they can also be employed in photovoltaics, as reported in different studies [44–46].

## 3. Experimental Section

Molecular calculations were carried out with the Gaussian09 code. In this study, we have tested the M06 family of density functionals [47–49] based on the ability to study properties of organometallic compounds; the hybrid functionals B3LYP [50–52] and PBE0 [53] have also been used in other studies demonstrating their predictive capacity. The basis sets used were Double Zeta Valence Polarization (DZVP) [54,55], Los Alamos National Laboratory 2 Double Zeta (LANL2DZ) [56–59] and split-valence Pople-type 6-31G(d) [60]. Table 5 shows the use of the basic sets for each of the atoms composing the molecular system.

Molecular structure calculations of the ground state were obtained by the established technique in Gaussian 09W. The strength constants and vibrational frequencies were determined via analytic frequency calculations on stationary points obtained after geometry optimization. Both calculations were carried out at the same theory level. Ultraviolet-Visible spectra (UV-Vis) were simulated using the TD-DFT approach; solvent effects sdfd taken into account by an implicit approach, namely the polarizable continuum model (PCM) [61], using the non-equilibrium version of the IEF-PCM model [62], with methanol being considered as solvent. The equations were solved for 20 excited states. The infrared (IR) and UV-Vis spectra were analyzed using the program SWizard [63,64].

In this work we calculated the total dipole moment (μ) and the isotropic polarizability (α). The molecular dipole moment is an experimental measure of the charge distribution in a molecule. It is difficult to accurately evaluate the global electron distribution in a molecule because it involves all the multipoles. The polarizability contributes in a significant way to the understanding of the response of the system facing an external field. On the other hand, using the DFT framework makes it possible to find the chemical reactivity descriptor values, such as electron affinity, ionization potential, electronegativity, hardness and electrophilic index. All these values were obtained using system energy calculations.

## 4. Conclusions

In this work, we studied a copper complex with a polypyridine-type ligand. A set of methodologies suitable for the study of this type of molecular systems was applied, and based on a comparison of experimental data with theoretical calculations, we elected the M06/LANL2DZ + DZVP level of calculation, which was found to be the better approach. The molecular system characterization includes the geometry optimization, the calculation of vibrations of functional groups, total dipole moment, isotropic polarizability, molecular orbitals, and chemical reactivity parameters. The molecular orbital energy indicates an energy gap of 3.146 eV in the Cu biquinoline dye. The influence of anion species such as hexafluorophosphate and chlorine does not contribute to the energy levels. Analyzing the data makes it possible to find potential applications for this dye in photovoltaic devices.

The M06/LANL2DZ + DZVP level of calculation can be used as a useful tool for studying the molecular structure and electronic properties of Cu biquinoline dye, as well as other structures derived from it.

## Figures and Tables

**Table 1:** Electron affinity and the ionization potential of Cu(I) biquinoline with different levels of theory.

Basis set	Functional	Electron affinity (eV)	Ionization potential (eV)
LANL2DZ + DZVP	M06	4.79	9.80
	M06-2X	4.65	9.93
	M06-HF	4.58	11.86
	M06-L	4.98	9.55
	B3LYP	4.88	9.84
	PBE0	4.79	9.79
LANL2DZ	M06	4.82	9.90
	M06-2X	4.64	10.01
	M06-HF	4.57	9.63
	M06-L	5.00	9.48
6–31G(d) + DZVP	M06	4.49	9.87
	M06-2X	4.26	11.01
	M06-HF	4.12	11.61
	M06-L	4.69	9.49

**Table 2:** Maximum absorption wavelength of Cu(I) biquinoline dye using various models.

Model chemistry	λ_max_ (nm)	Model chemistry	λ_max_ (nm)
M06/LANL2DZ + DZVP	556	M06-HF/LANL2DZ	282
M06-2X/LANL2DZ + DZVP	386	M06/LANL2DZ	543
M06-HF/LANL2DZ + DZVP	279	M06-2X/LANL2DZ	378
M06-L/LANL2DZ + DZVP	641	M06-L/6–31G(d) + DZVP	645
B3LYP/LANL2DZ + DZVP	614	M06-HF/6–31G(d) + DZVP	279
PBE0/LANL2DZ + DZVP	578	M06/6–31G(d) + DZVP	488
M06-L/LANL2DZ	629	M06-2X/6–31G(d) + DZVP	328

**Table 3:** Cu(I) biquinoline electronic excited states, showing wavelengths (nm), energies (eV), oscillator strength (f) and the orbitals involved in the transitions. Only excited states with oscillator strength > 0.02 are shown.

λ (nm)	*E* (eV)	Oscillator strength	Assignment; H = HOMO, L = LUMO, L + 1 = LUMO + 1, *etc.*
556.0	2.25	0.1652	H-1→L + 1(50%) H-0→L + 0(26%) H-1→L + 0(19%)
373.3	3.32	0.0308	H-3→L + 0(76%) H-2→L + 0(8%) H-4→L + 0(6%)
362.0	3.40	0.0372	H-3→L + 1(54%) H-2→L + 1(9%) H-5→L + 1(8%)
			H-4→L + 1(7%) H-1→L + 3(5%)
358.7	3.46	0.0225	H-6→L + 0(43%) H-10→L + 0(16%) H-2→L + 0(13%)
			H-9→L + 0(8%) H-5→L + 0(8%)
355.3	3.49	0.0295	H-5→L + 1(67%) H-5→L + 0(11%) H-3→L + 1(10%)
351.6	3.53	0.0392	H-6→L + 0(39%) H-10→L + 0(23%) H-9→L + 0(15%)
			H-2→L + 0(9%)

**Table 4:** Cu(I) biquinoline dye selected bond lengths (angstroms) and bond angles (degrees).

Parameters	Value	Parameters	Value
Cu1-N2	2.024	C50-O52	1.349
Cu1-N3	2.023	O52-H53	1.034
Cu1-N4	2.022	Cu1-N2-C28	126.0
Cu1-N5	2.025	Cu1-N2-C24	113.4
N4-C45	1.342	N2-Cu1-N5	121.4
N4-C41	1.374	N2-Cu1-N3	81.6
C45-C59	1.477	N2-Cu1-N4	128.5
C41-C42	1.437	N3-Cu1-N4	122.0
C44-H58	1.089	N3-Cu1-N5	128.2
C50-O51	1.245	N4-Cu1-N5	81.6

**Table 5:** Basis sets used in this study.

	Basis set	Atoms
Approach 1	LANL2DZ	C, H, O and N
	DZVP	Cu

Approach	2 LANL2DZ	C, H, O, N, and Cu

Approach 3	6–31G(d)	C, H, O and N
	DZVP	Cu
